# Composite Fibrin/Carbon Microfiber Implants for Bridging Spinal Cord Injury: A Translational Approach in Pigs

**DOI:** 10.3390/ijms241311102

**Published:** 2023-07-05

**Authors:** Alexandra Alves-Sampaio, Patricia Del-Cerro, Jorge E. Collazos-Castro

**Affiliations:** Neural Repair and Biomaterials Laboratory, Hospital Nacional de Parapléjicos (SESCAM), Finca La Peraleda S-N, 45071 Toledo, Spain; aalves@sescam.jccm.es (A.A.-S.); patriciadel.cerro.1@gmail.com (P.D.-C.)

**Keywords:** porcine, pig, spinal cord injury, contusion, compression, myelotomy, biomaterial, microfiber, conducting polymer, regeneration

## Abstract

Biomaterials may enhance neural repair after spinal cord injury (SCI) and testing their functionality in large animals is essential to achieve successful clinical translation. This work developed a porcine contusion/compression SCI model to investigate the consequences of myelotomy and implantation of fibrin gel containing biofunctionalized carbon microfibers (MFs). Fourteen pigs were distributed in SCI, SCI/myelotomy, and SCI/myelotomy/implant groups. An automated device was used for SCI. A dorsal myelotomy was performed on the lesion site at 1 day post-injury for removing cloths and devitalized tissue. Bundles of MFs coated with a conducting polymer and cell adhesion molecules were embedded in fibrin gel and used to bridge the spinal cord cavity. Reproducible lesions of about 1 cm in length were obtained. Myelotomy and lesion debridement caused no further neural damage compared to SCI alone but had little positive effect on neural regrowth. The MFs/fibrin gel implant facilitated axonal sprouting, elongation, and alignment within the lesion. However, the implant also increased lesion volume and was ineffective in preventing fibrosis, thus precluding functional neural regeneration. Our results indicate that myelotomy and lesion debridement can be advantageously used for implanting MF-based scaffolds. However, the implants need refinement and pharmaceuticals will be necessary to limit scarring.

## 1. Introduction

Biomaterials may provide mechanical support, spatial cues, and a favorable biochemical environment for growing neural cells, and therefore are becoming integral part of all advanced cellular and molecular strategies aimed at tackling spinal cord repair [1]. Numerous biomaterials have been implanted in preclinical models of SCI with the aim of providing scaffold to the regenerative neural tissue [2,3,4,5,6,7,8], support for transplanted cells [6,7,8,9,10], or delivery of growth factors and drugs [11,12,13]. Although the list of materials used is extensive, they mainly comprise polymers in the form of hydrogels, tubes, channels, fibrils, porous structures, or nanoparticles, and their combinations [1,2,3,4,5,6,7,8,9,10,11,12,13]. Because cell proliferation, growth, and differentiation, as well as neuronal activity, can be controlled electrically [14,15,16,17], electroconducting materials have also been proposed to fabricate active tissue scaffolds able to provide dynamical cues for neural repair [1,3,18,19]. Electroconducting MFs are especially suitable for this purpose because they promote long-distance, guided axonal growth [1,3,20,21], can be of any chosen length, and it is possible to place them parallel to the axons, thus minimizing tissue damage [3]. Moreover, they have no dead ends or empty reservoirs, thus reducing infection risks.

Carbon MFs have superior electrical and mechanical properties [22,23] that make them attractive for the fabrication of biomedical devices. To exploit their potential for neural repair, we coated 7-micron carbon MFs with the conducting polymer poly(3,4-ethylenedioxythiophene) doped with poly[(4-styrenesulfonic acid)-*co*-(maleic acid)] (PEDOT:PSS-*co*-MA), and subsequently with neural cell adhesion molecules that directly promoted neuronal attachment and axonal elongation [20], or with multimolecular complexes of growth factors and matricellular proteins that induced glial precursor cell migration, indirectly facilitating axonal elongation [21]. The latter biofunctionalized MFs were implanted in spinal-cord-transected rats, providing compelling evidence that they can enhance and guide blood vessel growth, cell migration, and axonal extension across the lesion [3]. Similar effects together with a prohealing immunomodulatory response were obtained in transgenic mice [24,25]. Remarkably, a single biofunctionalized microfiber was able to promote the adhesion and growth of numerous axons and glial cells both in vitro [20,21] and in vivo [3,24], with hundreds of axons following the pioneering ones in a process resembling developmental axon fasciculation.

Despite the compelling therapeutic results achieved in preclinical models, implantation of biomaterials in the injured spinal cord may produce further neural damage that must be clearly exceeded by their neuroregenerative effects to reach clinical application. Ideally, solid evidence of tissue repair with physiological and behavioral correlates of neural reconnection across the lesion should be demonstrated in large animal models of human neuropathology before advancing to human testing. In this regard, the use of swine in translational neuroscience is increasing because of its size, longevity, and developmental, physiological, and anatomical similarities [26,27,28,29] to humans. Biomaterials have been tested in pigs for reconstruction of different body organs [30,31,32,33], but their use in porcine spinal cord repair strategies is uncommon [4,13]. Nevertheless, several swine SCI models were developed in the last decade [4,13,26,34,35,36,37,38,39,40,41], and currently they enable a comprehensive testing of experimental therapies in a preclinical context that closely resembles the neuropathological features of human lesions.

Spinal cord contusion, compression, and laceration are the most frequent clinical presentations of traumatic SCI and cause liquefactive necrosis followed by neural tissue cavitation with variable grades of fibrosis [42,43]. Since the pioneering studies of Allen [44], numerous researchers have addressed the possible neuroprotective effects of performing an incision on the dura mater (durotomy) and the spinal cord (myelotomy) soon after the injury to relieve the increased intraspinal pressure and evacuate the necrohemorrhagic material [4,45,46]. The same surgical procedure has been advantageously used to implant biomaterials acutely at the lesion site to promote tissue repair [4,42,47]. Polymeric scaffolds were introduced in the contusion site in rats and pigs from 6 h to 3 days after injury [4]. Reduced spinal cord cavitation, accompanied by enhanced fibroblast, Schwann cell, and neurofilament-positive axon growth, was found in the rat lesion. Although histological results were not reported for pigs, these studies provided support to clinical trials that demonstrated safety of the implanted biomaterials in humans [42,47].

The aim of the present work was to investigate the feasibility and possible usefulness of implanting MF bundles in a clinically relevant porcine SCI model. For this, we produced contusion/compression SCI in pigs, obtained a baseline of histology and behavior, and studied the consequences of a dorsal myelotomy at one day post-injury, alone or followed by the implantation of biofunctionalized carbon MFs embedded in fibrin gel. Based on the abovementioned studies implanting those MFs in rodents [3,24,25], we considered that an implant with a small number of MFs (about one or two MFs per mm^2^ of transverse spinal cord section) might be sufficient to support collective cell regrowth after SCI in pigs. Besides characterizing the porcine SCI, we obtained preliminary evidence showing that the biofunctionalized MFs can also favor neural regrowth in this translational context. The results support the clinical usefulness of the MFs and provide guidance for further optimization of the implant, the surgical procedures, and the combinatorial treatment needed to achieve functional spinal cord repair.

## 2. Results

### 2.1. Biomechanical Parameters of the Injury

**Figure 1:**
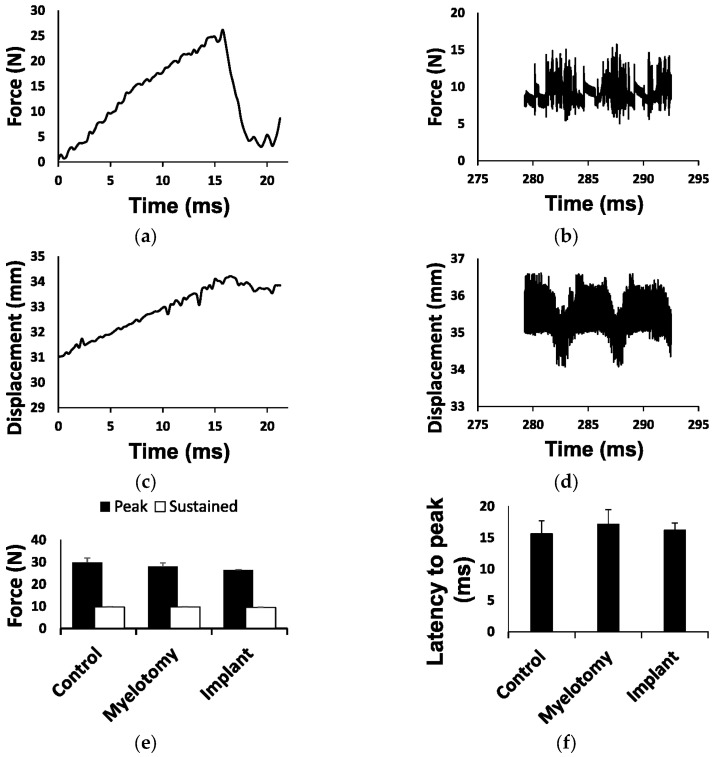

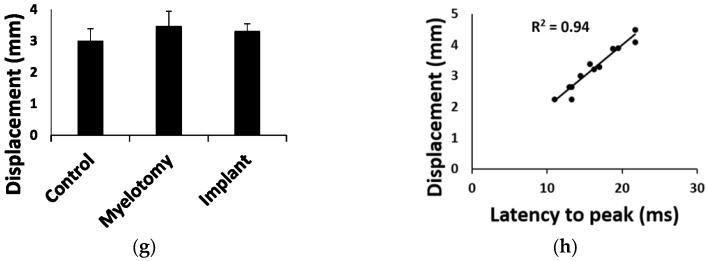
Biomechanical parameters of the injury. (**a**) Example of force development during the initial impact (contusion phase). The contact of the impactor with the spinal cord occurred at time = 0 and the peak force (set at 25 N) was reached about 16 ms later. (**b**) Example of force recorded during the compression phase. The average force was close to 10 N but oscillated between 5 and 15 N due to spinal cord movements with breathing. (**c**) Tissue displacement during the contusion phase (shown in (**a**)). (**d**) Tissue displacement during the compression phase shown in (**b**). Oscillations in force and displacement occurred in phase with respiratory movements. (**e**) Forces for each injury phase and treatment group. (**f**) Latency to peak force for each treatment group. (**g**) Tissue displacement for each treatment group. (**h**) Linear regression for latency to peak force and tissue displacement during the contusion phase, including data from all groups. Data in (**e**–**g**) are represented as the mean ± standard error.

The impact’s contusion peak force, sustained compression force, latency to peak, and spinal cord displacement were, on average, similar among the three experimental groups (Figure 1). In porcine SCI models using contusion alone [4,38], the mechanical ventilator can be stopped during the short time needed for the contusion (<30 s), thus reducing injury variability because of breathing movements without increasing tissue damage. However, in the current contusion/compression model, the total duration of the injury (>5 min) made it impractical to halt the machine. Although the spinal column was firmly fixed, the spinal cord itself moved with respiration, leading to some intersubject variability in the injury. The contusive peak force was reached about 16 ms after the impactor contacted the spinal cord (Figure 1a), and this time (latency to peak) varied from 11 to 22 ms without significant differences among groups (Figure 1f). During the initial impact, the spinal cord was displaced 2–5 mm (Figure 1g) depending on the phase of the respiratory movement, which also conditioned the latency to peak force; the longer the latency to peak, the higher the spinal cord displacement (Figure 1h). The impact’s peak force itself (Figure 1e) was similarly influenced and tended to be higher in the animals with less spinal cord displacement. On the other hand, the average force in the 5 min compression phase of the injury was close to the 10 N set for the three groups (Figure 1e), but it oscillated between 5 and 15 N (Figure 1b) due to the spinal cord displacements (Figure 1d) caused by respiratory movements.

### 2.2. Gross Histology

**Figure 2:**
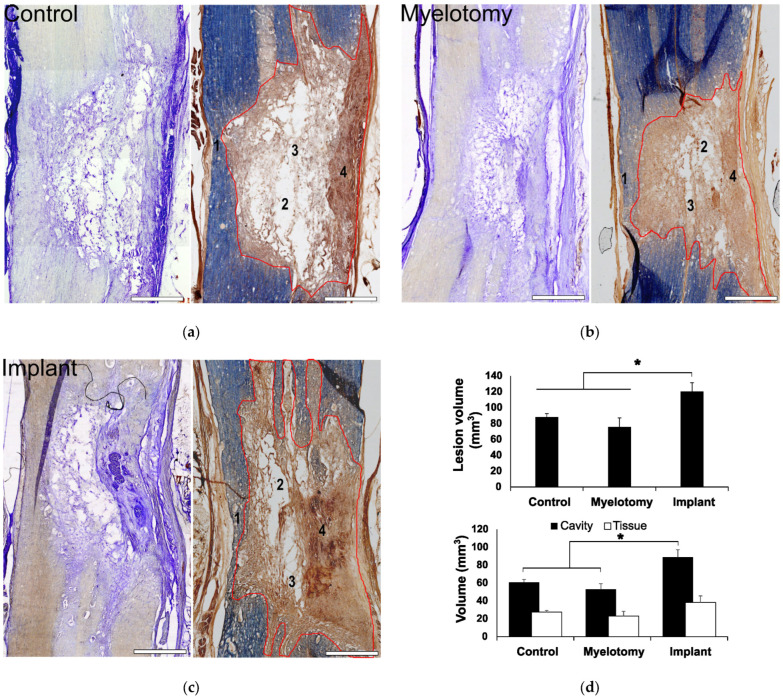
General assessment of the lesion at 30 days post-lesion (DPL). (**a**–**c**) 50 μm spinal cord sections from animals with control SCI (a), SCI + myelotomy (**b**), and SCI + myelotomy + implant (**c**), processed for cresyl violet (left) or eriochrome cyanine (right) staining. The lesion area is delineated in red. Numbers indicate the following: 1, spared white matter (blue) on one side of the spinal cord; 2, lesion cavities; 3, lesion trabeculae; and 4, dense meningeal scar in the most severely injured side. (**d**) Quantification of lesion volume (upper panel) and cavitation and tissue within the lesion for the different treatment groups. Data represent the mean ± standard error. Statistically significant differences are indicated with asterisks. * *p* < 0.05. Scale bar, 2 mm.

At the lesion epicenter, most of the spinal white and gray matter was destroyed in the control group, although a rim of uninterrupted white matter was frequently observed unilaterally in the ventrolateral region (Figure 2a). A cavity compartmentalized by scarring tissue formed at the lesion site. The scarring tissue had two differentiated zones (Figure 2a), namely, a compact part apparently arising from the meninges and present only in the side with complete interruption of the white matter, and a spongy part with cavities compartmentalized by trabeculae, likely originating from connective tissue of the spinal cord itself. Myelotomy and lesion debridement caused no further spinal cord damage; the gross histological features of the lesion (Figure 2b) were maintained with no significant differences in lesion volume, cavitation, or cellular content when compared to the SCI group (Figure 2d). In the implant group (Figure 2c), the lesion volume was enlarged together with cavitation and cellularity (Figure 2d; *p* < 0.01 compared to control SCI and myelotomy groups). Despite lesion enlargement, there was no significant increase in fibrosis, and a similar histological pattern of scarring with a compact and a spongy part was observed. Nevertheless, the implant increased axonal growth and glial migration into the lesion, as demonstrated by immunohistochemical studies.

### 2.3. Immunohistochemistry

**Figure 3:**
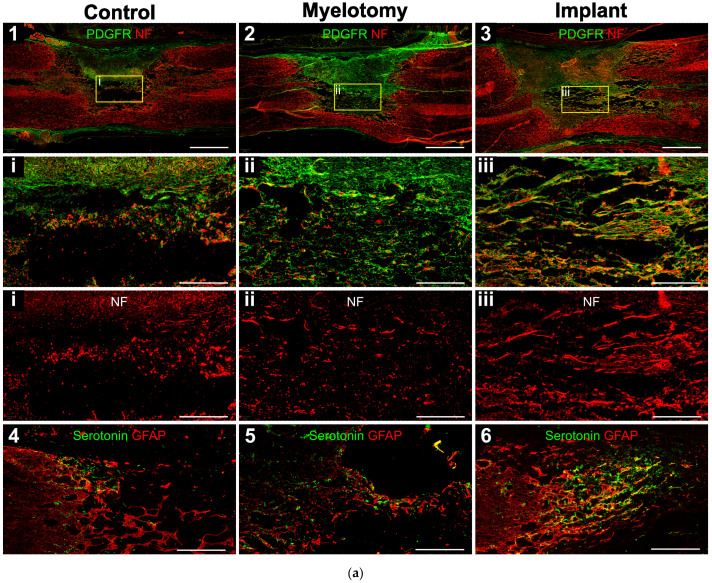

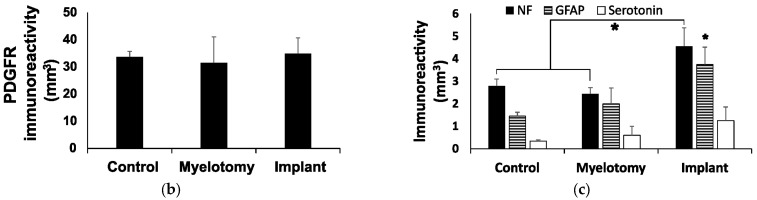
Immunohistochemical assessment of the lesion at 30 DPL. (**a**) 10-μm spinal cord sections representative for the different treatment groups, immunostained for the cell markers indicated in the panels. 1–3: Double fluorescent staining for fibrotic tissue (PDGFRβ, green) and axons (neurofilament, NF, red). The regions in squares are magnified below, including a view of only NF for a better appreciation of axons within the lesion. Note that in the control case, most NF-positive cellular processes are fragmented axons not yet removed from the lesion at the time of tissue fixation, whereas in the implanted animal, numerous axons grew associated with migrating PDGFRβ-expressing cells. 4–6: Spinal cord sections showing double immunostaining for serotonergic axons (green) and astrocytes (GFAP, red). The rostral border of the lesion is on the left. (**b**,**c**) Quantification of stained cellular processes for the different treatment groups. Data represent the mean ± standard error. Statistically significant differences are indicated with asterisks.* *p* < 0.05. Scale bars: 1–3, 2 mm; square magnifications in i–iii, 500 μm; 4–6, 500 μm.

Fibrosis was assessed by immunostaining with an antibody against PDGFRβ that efficiently labels scarring fibroblasts and pericytes in rodent models of spinal cord [3] or brain [48] injury. The combination of PDGFRβ and neurofilament (NF) immunostaining allowed for a clear visualization of the borders of the lesion and analysis of the scarring and neural growth responses into the lesion (Figure 3).

Like in rodents, [3] we found that the fibrotic tissue at the SCI site was strongly positive for PDGFRβ in pigs (Figure 3a, panels 1–3), with no statistical differences among groups regarding the lesion volume occupied by PDGFRβ-positive cells (Figure 3b). The compact part of the scar was essentially impenetrable to axons and astroglia irrespective of the treatment group; however, neural cell growth in the spongy part was substantially different. In control SCI animals, the spongy scar contained mostly fragmented axons (Figure 3a, panel 1) that were presumably injured by the original trauma and were not yet removed by phagocytic cells. Serotonergic axons generally remained at the rostral border of the injury and did not enter the lesion (Figure 3a, panel 4), whereas some astrocytes penetrated for about 500 μm.

**Figure 4:**
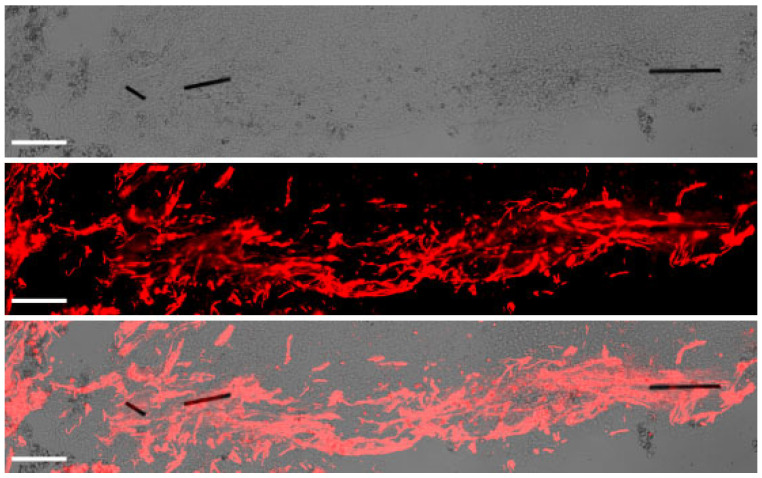
Axonal growth and guidance supported by microfibers (MFs) implanted in the porcine spinal cord. Transmitted light imaging (**top**) enabled visualization of the MFs, whereas fluorescent immunostaining for neurofilament (red, **middle**) allowed the identification of axons. The merged image at (**bottom**) illustrates fasciculated axons penetrating the lesion aided by the MFs, which effectively bridged the spinal cord cavity. Scale bar, 100 μm.

Myelotomy alone was not efficient to induce neural growth, although fewer fragmented axons were observed in the trabecula within the lesion, compared to control SCI, and some serotonergic axonal sprouts crossed the proximal lesion border and entered with astrocytes for about 1 mm (Figure 3a, panels 2 and 5). On the contrary, abundant axons elongated within the lesion in the implant group and aligned with the longitudinal axis of the spinal cord (Figure 3a, panel 3). Numerous axons grew associated with the MFs (Figure 4), forming small fascicles that extended for several millimeters, as previously reported in experiments with rodents in vitro [20,21] and in vivo [3,24], and confirming that the biofunctionalized MFs can bridge large spinal cord lesions of clinical relevance. The implant also enhanced the sprouting of serotonergic axons and their growth associated with astroglial cells migrating into the lesion (Figure 3a, panel 6), although those axons still stopped at about 1 mm from the border, presumably inhibited by the PDGFRβ+-fibrotic scar.

### 2.4. Animal and Behavioral Outcomes

No deaths associated with SCI or treatment were recorded. SCI invariably produced urinary retention, and therefore the urethral catheter was kept in place for voiding until the micturition reflex recovered by 4–6 DPL. The animals were essentially paralyzed and dragged the hindlimbs on the ground, becoming prone to skin pressure ulcers over bony prominences in the hindlimbs. Tissue healing ointments and massages were applied on the damaged skin, with the hindquarters of the animal raised from the ground using harnesses or wheelchairs. Two animals (one in the SCI group and other in the implant group) had rectal prolapse after 20 DPL that was effectively treated with a tobacco-pouch suture. No other complications arouse.

**Figure 5:**
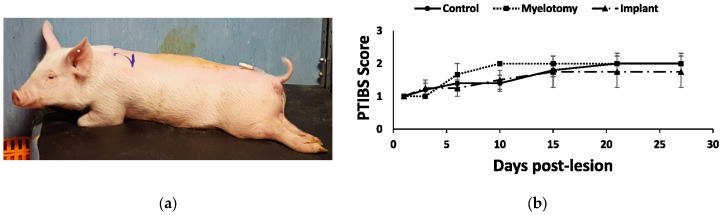
Behavioral outcomes after SCI. (**a**) Photograph at 1 DPL illustrating the typical posture of the animals with the paralyzed hindlimbs. (**b**) Behavioral recovery as assessed using the PTIBS scale [36]. There was little recovery with no significant differences between the treatment groups during the four weeks of follow-up.

Because the injury damaged most of the spinal cord in the transverse plane, the animals showed a severe neurological impairment with little or no improvement during the month of follow-up (Figure 5). At 28 DPL, all groups of pigs remained essentially paraplegic, as reflected by a score of 1 (no active hindlimb movements) or 2 (active hindlimb movements, with rump and knees on the ground) out of 10 points when assessed with the PTIBS scale [36].

## 3. Discussion

Treatments that promote clinically meaningful spinal cord regeneration remain an unmet challenge. Assessing neurotherapeutics in a preclinical context resembling the human condition will likely allow for a better identification and refinement of treatments deserving of testing in clinical trials. Here, we developed a contusion/compression SCI model in swine and obtained preliminary evidence that electroconducting MFs can favor neural regrowth in this model when embedded in fibrin hydrogel and implanted at 1 DPL. Several biomaterials have been assessed in pigs for repair or replacement of organs different to the brain and spinal cord [30,31,32,33]. Moreover, the distribution of nanoparticles aimed at drug delivery has been studied in a contusion model of porcine SCI [13], as well as the feasibility of introducing scaffolds made of a copolymer of lactic-co-glycolic acid (PLGA) and poly(L-lysine) into the lesion [4]. Biofunctionalized electroconducting MFs have been shown to enhance and guide axonal and blood vessel growth after SCI in rodents [3,24,25] and can be advantageously used for sensing [49] and stimulating [50] neuronal activity. To our knowledge, the present study represents the first attempt to promote spinal axon regeneration in a large animal by implanting an MFs-based electroconductive scaffold. Although the implant design and surgical procedures still need optimization to prevent lesion enlargement and achieve functional spinal cord repair, the results support the possible clinical benefit of performing early myelotomy, lesion cleaning, and implantation of MFs/fibrin gel bundles in human SCI.

In average, human spinal cord lesions extend longitudinally for about 2 cm [51,52] but range from a few mm to 10 cm or more. Handling single, long carbon MFs and keeping their alignment within a large lesion is an extremely laborious task. To be clinically useful, they must be conveniently presented as a bundle within a gel suitable for implantation. Fibrin has a crucial role in blood clotting and wound healing, and fibrin hydrogels have demonstrated safety and usefulness in SCI repair strategies [53,54]. Like our previous studies in mice [25], embedding multiple MFs in fibrin facilitated their handling, alignment, and implantation in the porcine spinal cord. Additionally, fibrin partially filled the tissue gap, thus reducing accumulation of blood products in the cavity after myelotomy and lesion debridement. However, the fibrin/MFs implant enlarged the spinal cord lesion, probably by increasing intraspinal pressure [4,46] when the meninges were sutured after the introduction of an excessive number of fibrin/MF bundles. Several strategies may be implemented to prevent this adverse effect, such as using smaller gel cylinders, fibrin gelification directly within the cavitated tissue, expansive duroplasty, and co-administration of medications. Performing MRI examinations of the lesion before and after the surgical treatment may also help to identify the optimal size and geometry of the implant for each individual case [6], as well as to investigate axonal damage and regeneration in the spinal cord [55], thus reducing the risks of further tissue damage while improving the neuro-reparative effects.

Spinal cord contusion followed by sustained compression is a frequent clinical presentation of human traumatic SCI and was modeled in swine [36], finding that the combined injury produced greater tissue damage and functional impairment than contusion alone. In the present work, the force parameters were chosen to cause severe spinal cord damage. Although the lesion was almost complete in the transverse plane, a rim of white matter was usually preserved in the ventrolateral region of one side, probably due to subtle spinal cord displacement during the contusion/compression procedure. The completely damaged side always exhibited a greater extent of fibrosis, with a solid mass of cells arising from the meninges and penetrating the periphery of the lesion. It is likely that a greater force concentration in that side also disrupted the pia matter, leading to meningeal scarring that, in absence of preserved white matter, invaded the lesion.

Differently to the dense meningeal scar, which was largely impenetrable to axons and glial cells in all treatment groups, the spongy scar contained numerous longitudinally aligned axons in the implant group. Thus, as previously demonstrated in rodents [3,24,25], the multimolecular complex of PLL/heparin/bFGF/fibronectin attached to the MFs was able to facilitate the growth of some axonal types in the porcine spinal cord, even in the inhibitory environment provided by the scarring cells. Neural tracers can be applied in swine [26,29] to study the origin and termination of spinal axonal tracts. However, in this preliminary work, only immunohistochemistry was used for axonal labeling, and no precise identification of the neuronal sources of regrowing axons was achieved. Some of the neurofilament-positive axons extending with migratory cells along the MFs likely originated from propriospinal neurons able to regenerate spontaneously after SCI [56]. In this regard, our results are in line with studies in rodents showing that some growth factors can transform the fibrotic scar into a friendlier substrate for propriospinal axons by increasing the expression of extracellular matrix molecules [57].

Enhanced sprouting of serotonergic axons was detected in the rostral border of the lesion in response to the implant, although those axonal sprouts were still unable to elongate across the entire length of the lesion. Serotonergic axons innervating the spinal cord originate from neurons located in the caudal brainstem, mostly in the midline raphe nuclei of the medulla oblongata and some parts of the reticular formation [58,59]. They have a facilitatory role in locomotion and postural movements, and participate in autonomic and sensory regulation [58,59]. In fact, administering serotonin agonists or intraspinal grafts of serotonergic neurons improves motor performance after SCI [58]. Our present findings in pigs agree with studies in rodents showing that the sprouting of serotonergic axons can be enhanced by different molecular and cellular interventions [59], including the implantation of PLL/heparin/bFGF/fibronectin-coated MFs [3]. Serotonergic neurons are more efficient at sprouting compared to cortical neurons [3,60]. Whether their axons are less sensitive to the effects of the inhibitory environment at the lesion is unclear; however, they exhibit reduced retrograde degeneration after axotomy, maintain a more active growth cone, and have increased growth associated protein-43 (GAP-43) and β1 integrin expression [60], which may allow them to grow better in lesion environments containing extracellular matrix proteins such as laminin or fibronectin.

In pigs receiving the MFs implant, serotonergic axons sprouted and penetrated the rostral border of the lesion intimately associated with migrating astrocytes. Although astroglial cells have been historically considered inhibitory for axon regeneration [61], their positive role in brain and spinal cord repair is currently widely recognized [4,62,63]. In fact, the presence of aligned astrocytes migrating into the spinal cord lesion is associated with much greater numbers of axons regenerating from supraspinal systems in response to molecular treatments or biomaterials [6,64,65]. Moreover, our studies in vitro showed that axonal elongation on the MFs coated with PLL/heparin/bFGF/fibronectin is strictly dependent on the migration of permissive cells, particularly glial progenitor cells [21]. Taken together, these studies suggest that increasing astrocyte migration into the lesion will further enhance axonal regeneration and, likely, functional recovery after SCI.

The contusion/compression injury destroyed most of the grey matter and white matter in the transverse aspect of the porcine spinal cord, causing severe motor deficits with little or no recovery in the four-week follow-up period. The functional outcomes are comparable to those reported for Yucatan miniature pigs subjected to a combined contusion/compression SCI at T10/T11 [36], and to those resulting from severe midthoracic contusion SCI in rats, which are essentially due to the interruption of spinal axonal tracts [66]. Nevertheless, we cannot completely rule out that local neuronal death at the injured thoracolumbar segment of the porcine spinal cord had contributed, although to a lesser degree than axotomy, to the severity and chronicity of motor dysfunction. Motoneuron death caused by contusion at the cervical or lumbar spinal cord enlargement is associated with permanent locomotor impairments in rats [67], and it is possible that a small part of the motoneurons innervating low back muscles [68] and hip muscles [69] died in our porcine SCI model. Additionally, some neurons of the locomotor central pattern generator [70] were likely present at the injured spinal cord segment and became damaged, further worsening the functional outcome.

In agreement with the insufficient histological repair of the spinal cord, the implant had no evident benefits on motor recovery. Again, this is consistent with studies in rodents showing that propriospinal axon regrowth across a thoracic spinal cord lesion facilitated by a combination of biomolecules and biomaterials has no significant impact in motor behavior in the month following the lesion [57]. Presumably, some functional axonal reconnection across the lesion will be detected in a long-term follow-up using electrophysiological techniques, although behavioral recovery will still be limited.

To achieve clinically relevant neural repair, several features of the implant and the surgical procedure need refinement. First, further tissue damage must be avoided by using the appropriate number, size, and placement of MFs/fibrin gel bundles that best match the geometry of the cavity without compressing undamaged tissue or increasing intraspinal pressure (ISP). Monitoring ISP [4,46,71] may be necessary to rule out this possibility and optimize the surgical methodology. Second, additional interventions are needed to control fibrosis. Debriding the lesion and implanting MFs/fibrin gel bundles was ineffective in preventing accumulation of fibrotic tissue at the spinal cord cavity. The scarring tissue seemed to originate from meningeal cells and intraspinal connective tissue as proposed by others [72,73,74]. However, the most compact part of the scar, largely impenetrable to axons, apparently arouse from the meninges and needs further attention. Although inhibitory to supraspinal serotonergic axons, the spongy part of the scar in the “implant group” contained numerous axons surrounded by PDGFRβ-positive cells, suggesting that this part of the scarring tissue can be turned into a supportive cell environment for some axonal types [57]. Finally, the proposed treatment must be complemented with electrical stimulation (ES) or pharmaceuticals to promote supraspinal axonal regeneration. Suppression of PTEN (phosphatase and tensin homolog), a negative regulator of the mammalian target of rapamycin (mTOR) pathway, enhances regeneration of the corticospinal tract after SCI [75]. The same biochemical pathway is modulated in corticospinal neurons by ES applied at the cerebral cortex, inducing sprouting of intact corticospinal axons [76]. Therefore, our current working hypothesis is that ES through the implanted MFs will further enhance neural regeneration and synaptic reconnection in the injured spinal cord.

## 4. Materials and Methods

### 4.1. Impactor Device

**Figure 6:**
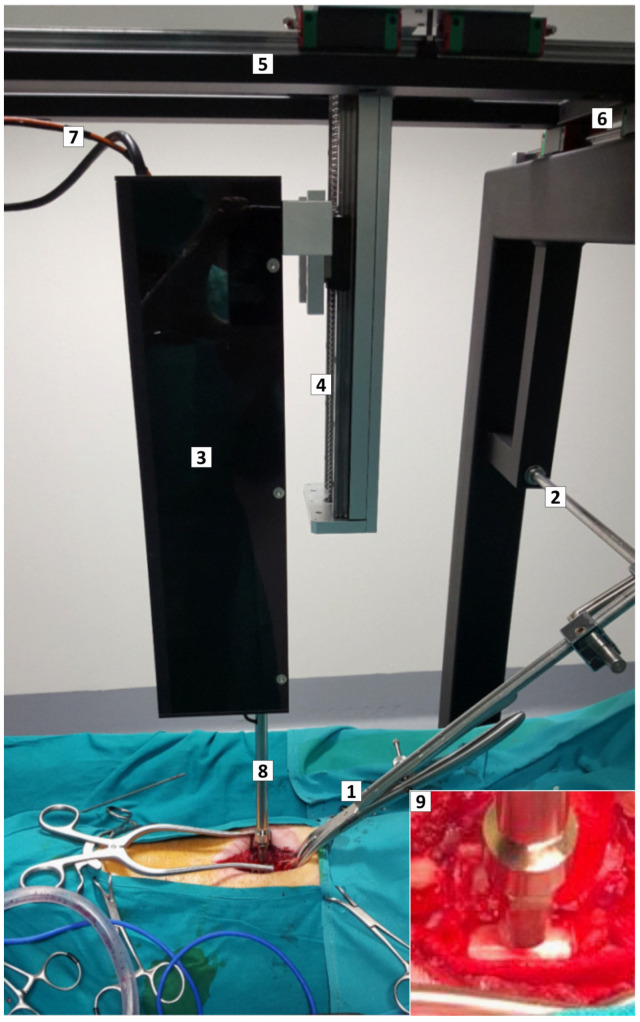
Custom-designed impactor device and positioning framework. Intraoperative photograph illustrating the device and the framework with the pig ready for SCI. (**1**,**2**) Forceps assembled on a track to immobilize the spine. (**3**) Box containing the stepping motor, the position sensor, and the impactor rack. The box is mounted on a rail with movement on the X, Y, and Z axes (**4**–**6**). (**7**) Cables communicating the sensors and the motor with the controller. (**8**) Rack with the force sensor and impacting tip, aligned to the spinal cord. (**9**) Magnified view of the force sensor and impacting tip (8 mm diameter flat circular head) close to the porcine spinal cord.

A motorized, servo-controlled impactor was developed for contusion/compression of the porcine spinal cord (Figure 6). The device allowed accurate control of the force, speed, and duration of the impact. The geometry of the impactor’s tip (8 mm diameter flat circular head) was aimed at producing lesions of about 1 cm in length. The impact sensor had a dynamic response and the applied force, ranging from 0 to 100 N, could be measured continuously with a precision of 99.8%, making possible the adjustment of the compressive force in presence of respiratory movements. Force and displacement data were recorded for analysis.

### 4.2. Animals and Experimental Groups

Fourteen 2-month-old Large White female pigs (*Sus scrofa domesticus*) weighing 10–15 kg at the time of lesion were used. The animals were purchased from a commercial supplier (Granja Agropardal, Toledo, Spain) and were randomly assigned to three experimental groups: (1) SCI (n = 5); (2) SCI/myelotomy at 1 DPL (n = 4); (3) SCI/myelotomy/implant (n = 5). For simplicity, these groups are referred to as SCI, myelotomy, and implant, respectively.

### 4.3. Anesthesia and Surgical Procedures

All surgical procedures were performed under inhalational anesthesia. Anesthesia was induced by intramuscular (IM) injection of ketamine (10 mg/kg), midazolam (0.1 mg/kg), and medetomidine (0.02 mg/kg), followed by intravenous (IV) administration of propofol (3 mg/kg). Then, a tracheal tube was placed, and the anesthesia was maintained with sevoflurane (1.7–2%) together with remifentanil (26 mg/kg/h IV) and rocuronium (1.2 mg/kg/h IV). Mechanical ventilation (Fabius Tiro, Dräger, Lübeck, Germany was set at 12–14 breaths/min with a tidal volume of 10–15 mL/kg. Heart rate, blood pressure, exhaled carbon dioxide, blood oxygen saturation, and inspired and expired sevoflurane levels were monitored (Infinity Delta, Dräger, Lübeck, Germany). A urethral catheter was inserted to prevent urinary retention.

With the animal in prone position on the operating table, an electrocautery pen was used to expose the vertebral spinous processes, dorsal laminae, and transverse processes from T12 to L2. The last thoracic vertebra (usually T14) was identified by the insertion of the last rib and submitted to dorsal laminectomy avoiding damage to the dura mater. A bone defect of at least 1 cm in diameter was created to avoid collision of the impactor with the vertebra. The vertebra immediately rostral was clamped to reduce spine movements associated with breathing, and the impactor was centered on the dorsal spinal cord midline. All animals received the same thoracolumbar lesion consisting of a contusion impact at 0.2 m/s with a nominal peak force of 25 N followed by 5 min compression at 10 N. The parameters for the combined contusion/compression injury aimed at producing a severe lesion with permanent paraplegia and were informed by data from Lee et al. [36], who showed that porcine spinal cord contusion impacts of about 20 N allowed for substantial walking recovery, while adding a 5 min compression force after the contusion substantially worsened the functional outcome. The lesion was inflicted at the thoracolumbar junction because this region is frequently injured in humans, only behind cervical injuries, whereas lesions at midthoracic segments are relatively rare [42].

**Figure 7:**
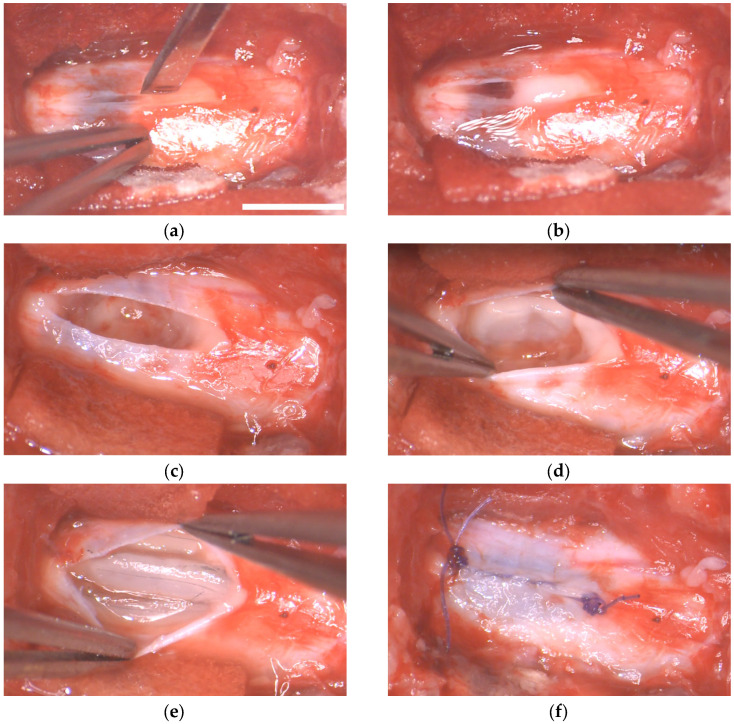
Implantation of fibrin/MFs bundles in the porcine spinal cord. Intraoperative photographs of the surgical procedure. (**a**) Dorsal view of the lesion site at 1 DPL. A durotomy is performed in the midline. (**b**) Hemorrhagic liquid and devitalized tissue protrudes through the incision of the dura matter. (**c**) Aspect of the cavity after irrigating the lesion with saline solution and removing blood clots and tissue adherences. (**d**,**e**) Accommodation of fibrin/MFs bundles into the cavity. (**f**) Suture of the dura mater. Scale bar, 5 mm.

Myelotomy and implant were successfully performed at 1 day post-lesion (DPL, Figure 7). In “myelotomy” and “implant” groups, the lesion site was exposed, and the meninges were incised in the dorsal midline. This allowed some of the liquefied necrohemorrhagic material to be extruded spontaneously from the lesion. The remaining devitalized tissue, clots, and tissue adherences could be removed using saline solution irrigation and tweezers, rendering a relatively clean cavity at the lesion site. No additional procedures were performed on the tissue defect in the myelotomy group. In the implant group, fibrin cylinders of ~1 mm in diameter and 5 to 10 mm in length containing the MFs were accommodated in the irregularly shaped cavity. Thirty MFs were implanted per animal.

All animals received postoperative treatment with meperidine (4 mg/kg, SC) every 12 h for 2 days for pain, marbofloxacin (2 mg/kg, IM) for 7 days as antibiotic, and meloxicam (0.2 mg/kg) subcutaneous (SC) for 7 days as anti-inflammatory agent.

### 4.4. Preparation of MFs/Fibrin Implants

Poly(3,4-ethylenedioxythiophene) doped with poly[(4-styrenesulfonic acid)-*co*-(maleic acid)] (PEDOT:PSS-*co*-MA) was electropolymerized on carbon MFs (7 μm diameter, Goodfellow, Huntingdon, UK) applying a constant current of 1 μA/mm^2^ and a charge density of 192 mC/cm^2^. The conducting polymer-coated MFs were sterilized with formaldehyde gas at 60 °C before their functionalization. The detailed protocol of microfiber preparation and physicochemical characterization, and their biofunctionalization with a multimolecular complex of poly-L-lysine (PLL), heparin, basic fibroblast growth factor (bFGF), and fibronectin, has been detailed in previous publications [3,21]. In brief, the first molecule (PLL) was covalently bonded to the carboxylic groups of PEDOT:PSS-*co*-MA. Subsequently, 10 mM heparin (Sigma, St. Louis, MO, USA; H5515) dissolved in PBS was applied for 4 min. Then, recombinant human bFGF (PeproTech, London, UK; 100-18B) was applied at 1 μg/mL in PBS for 1 h. Finally, the MFs were incubated for 4 days at 37 °C in PBS containing 40 mg/mL bovine fibronectin (Invitrogen, Waltham, MA, USA; 33010-018).

Fibrin was gelified on serial layers of MFs by enzymatically cleaving fibrinogen with thrombin in the presence of calcium ions and factor XIII. For this, two solutions (namely, A and B) were prepared. Solution A contained human fibrinogen (Sigma Aldrich, St Louis, MO, USA; F4883; 133 mg/mL) and factor XIII (CSL Behring, King of Prusia, PA, USA; Cluvot; 24 U/mL) in saline solution, whereas solution B had thrombin (Sigma Aldrich, St Louis, MO, USA; T7009; 24 U/mL) and 8 mM calcium chloride in saline solution. The gels were generated by mixing solutions A and B at a 2:1 ratio.

### 4.5. Animal Care

After arrival to our facilities, the pigs were housed in groups of two at a 12 h light/dark cycle, and were allowed to acclimate to the new environment for at least 2 weeks prior to SCI. They were fed twice a day and water was available *ad libitum.* They also had access to some toys. The person responsible for behavioral assessment spent at least two hours per day with the pigs to habituate them to human contact. After SCI, the pigs were housed individually, and the pens were adapted with mats to prevent skin sores. To avoid infections, urinary catheters were plugged, and the bladders were manually emptied with a syringe coupled to the catheter three times a day.

### 4.6. Motor Assessment

Functional recovery was assessed at 1, 3, 6, 10, 15, 21, and 28 DPL using the Porcine Thoracic Injury Behavior Scale (PTIBS) [36]. The PTIBS is a 10-point scale that describes the progression in hindlimb function from no active hindlimb movement (score 1) to apparently normal ambulation (score 10). The scale can be divided into three parts, with scores from 1 to 3 describing dragging, scores from 4 to 6 corresponding to stepping, and scores from 7 to 10 indicating walking behavior. Motor behavior was assessed by 2 independent evaluators, who were previously trained for administering the PTIBS scale, until their scores coincided in more than 90%. In case of disagreement on some experimental data, the scores from both evaluators were averaged.

### 4.7. Histological Procedures and Analyses

At 30 DPL, inhalational anesthesia was administered, and three spinal cord segments rostral and caudal to the lesion were surgically exposed before killing the animals with pentobarbital (120 mg/kg IV). The exposed segments were extracted within 3 min after death of the animal. For this, the spinal cord was transected with scissors in the rostral limit, the dura mater was held with tweezers, and the cut border of the spinal cord was progressively elevated from the vertebral channel while simultaneously cutting all nerve roots and ligaments to release the tissue. When the caudal limit was reached, the spinal cord was transected again and the tissue was collected and immediately immersed in 4% formaldehyde in 0.1 M, pH 7.35 phosphate buffered saline (PBS), for 4 days. This time of fixation is sufficient for formaldehyde penetration in large tissue specimens [77] and reaction with amino acids [78], producing excellent results in immunohistochemistry [26,29] while avoiding the negative effects of over fixation. The formaldehyde solution was replaced at 24 h to remove blood clots. After fixation, each spinal cord segment was separately cryoprotected by immersion in 30% sucrose for 4 days at 4 °C and embedded in optimal cutting temperature (OCT) matrix. The tissue was stored in OCT at −20 °C until cut in a cryostat.

Histological analyses were performed in horizontal serial tissue sections, separated 480 μm in the dorsoventral plane of the spinal cord, and cut at 10 μm thickness for immunohistochemistry or at 50 μm for eriochrome cyanine and cresyl violet stainings. Between 11 and 13 sections spanning the entire dorsoventral plane of the injured spinal cord segment were studied for each animal. For immunohistochemistry, tissue sections were blocked for 1 h at room temperature (RT) in PBS containing 1.2% triton and 2% normal goat serum, rinsed three times with PBS, and then incubated overnight at 4 °C with the primary antibodies neurofilament (NF, Sigma Aldrich, St Louis, MO, USA; N0142, 1:500), serotonin (SER, ImmunoSolution-IG1112, 1:2500), glial fibrillary acidic protein (GFAP, BD Biosciences, San Jose, CA, USA; 556327, 1:500), or platelet-derived growth factor receptor beta (PDGFRβ, Abcam, Cambridge, UK; AB32570, 1:100). On the next day, the sections were incubated for 2 h at RT in a PBS solution containing donkey secondary fluorescent antibodies (Alexa Fluor anti-rabbit 488, Alexa Fluor anti-mouse 594; Abcam 1:1000). In addition, cell nuclei were labeled with Hoechst 33342 (Molecular Probes, Eugene, OR, USA; 1.5 mg/mL in PBS applied for 15 min).

The lesion was initially visualized in mosaic images of eriochrome cyanine or cresyl violet stained sections obtained at 2776 × 2074 pixels with a stereology BX61 microscope (Olympus, Tokyo, Japan) equipped with a 4× objective. Subsequently, lesion volume, tissue cavitation, fibrosis (PDGFRβ^+^ tissue), and growth of axons (NF, SER) and astrocytes (GFAP^+^ cells) into the lesion were quantified on immunostained sections. For this, mosaic images (about 3 cm^2^) of the 10 μm fluorescent tissue sections were captured with an Olympus 1X83 microscope equipped with a 10× objective and a digital camera (Orca-Flash 4.0) controlled by the CellSens Dimension software 4.1. Mosaics were imported into the QuPATH software 0.4.3 to define the regions of interest (ROI, i.e., the lesion), which were further processed with the ImageJ software 1.53 for quantifications. The damaged area measured in each serial image was used to calculate lesion volume, which resulted from multiplying the dorsoventral diameter of the spinal cord by the average value of lesion areas. In the ROIs, the lesion area devoid of cellular elements (i.e., cavitation) was also measured and interpolated to lesion volume. Subtracting cavitation volume from total lesion volume resulted in the volume of tissue occupying the lesion. Specific cellular elements in that tissue (fibrosis, axons, astrocytes) were quantified in a similar way, based on the immunoreactive fluorescent area for the respective cellular markers as indicated before.

### 4.8. Statistical Analysis

Statistical analyses were performed with SigmaStat 9.0. All values reported, unless otherwise stated, are means ± standard error of the mean (SEM). All data groups were examined for normality using the Kolmogorov Smirnov test. One-way or two-way ANOVA followed by the Holm Sidak posttest were used to compare the average values of the biomechanical parameters of the injury and the histological measurements. Differences were considered statistically significant at *p <* 0.05. Linear regression was performed to model the relationship between contusion peak force and spinal cord displacement.

## Data Availability

The data presented in this study are available on request from the corresponding author.
